# Monkeypox and HIV in the Canary Islands: A Different Pattern in a Mobile Population

**DOI:** 10.3390/tropicalmed7100318

**Published:** 2022-10-19

**Authors:** Christian Betancort-Plata, Laura Lopez-Delgado, Nieves Jaén-Sanchez, Tomás Tosco-Nuñez, Laura Suarez-Hormiga, Carmen Lavilla-Salgado, Elena Pisos-Álamo, Araceli Hernández-Betancor, Michele Hernández-Cabrera, Cristina Carranza-Rodríguez, Marta Briega-Molina, José-Luis Pérez-Arellano

**Affiliations:** 1 Unidad de Enfermedades Infecciosas y Medicina Tropical, Hospital Universitario Insular de Gran Canaria, 35016 Las Palmas de Gran Canaria, Spain; 2 Servicio de Microbiología y Parasitología, Hospital Universitario Insular de Gran Canaria, 35016 Las Palmas de Gran Canaria, Spain; 3 Departamento de Ciencias Médicas y Quirúrgicas, Universidad de Las Palmas de Gran Canaria, 35016 Las Palmas de Gran Canaria, Spain

**Keywords:** monkeypox, human immunodeficiency virus, Canary Islands, Spain

## Abstract

Background. The clinical and epidemiological data of the recent outbreak of monkeypox (MPX) differ from previous reports. One difference is the epidemiological profile; the disease mainly affects a subgroup of MSM (men who have sex with men) with high-risk sexual behaviors, frequently persons living with human immunodeficiency virus (PLHIV). Methods. In this observational analysis, all patients with PCR (polymerase chain reaction)-confirmed MPX attending an Infectious Diseases and Tropical Medicine Unit in Gran Canaria (Spain) between May and July 2022 were considered. Results. In total, 42 men were included; 88% were identified as MSM, with a median age of 40 years. Only 43% were born in Spain. All the patients had systemic symptoms and skin lesions. The distribution of lesions was more frequent in the genital/anal region, and the involvement of hands and feet was less common. Fever and lymphadenopathies were less frequent than in other series. Other unusual manifestations were proctitis, pharyngitis and penile–scrotal edema. Half of the patients had other associated infections (mainly STIs, sexually transmitted infections), and 60% of the monkeypox patients had PLHIV (People Living with HIV). When comparing the clinical characteristics between HIV-positive and -negative patients, we found three main differences: (i) a higher frequency of perioral lesions, (ii) a higher frequency of pharyngitis and (iii) a higher number of sexually transmitted infections in HIV-positive patients. Conclusions. The clinical findings in this outbreak of MPX had great variability in presentation. Several clinical differences were found in PLHIV-coinfected patients.

## 1. Introduction

Monkeypox is a disease caused by a double-stranded DNA virus belonging to the *Poxviridae* family, *Chordopoxvirinae* subfamily, and *Orthopoxvirus* genus [1,2]. Its name derives from the fact that it was initially isolated in two outbreaks of pustular lesions in laboratory macaques in Denmark in 1958 [1]. In 1970, the disease was described in humans in Zaire (now the Democratic Republic of the Congo) [1,2,3], and it is considered a zoonosis that can affect multiple species of small mammals [4]. Since then, and until 2022, multiple human cases of the disease have been reported in two different patterns: endemic (in Central and West Africa) and those exported from these regions to developed countries [1,2,3]. On 6 May 2022, a case of monkeypox was identified in a UK resident associated with travel to Nigeria, which is considered the origin of the current outbreak of this disease [5]. Since then, monkeypox disease in humans has been reported in more than 86 developed countries, with Europe being one of the most frequently affected areas [6]. Most cases of this outbreak, technically a pandemic, have been reported in men who have sex with other men (gay and bisexual population) [7,8,9,10,11,12,13,14,15]. Spain is one of the countries with the highest number of reported cases in the world, and the Canary Islands, in particular, are one of the regions with the highest proportion of cases per number of inhabitants [16]. Some areas in the south of Gran Canaria are common tourist destinations for the European and Latin American LGTBI (lesbian, gay, bisexual, transgender, and intersex) community. In fact, one of the possible points of origin and focus of the current outbreak of monkeypox was the Gay Pride festival in Gran Canaria (5–15 May 2022), which was attended by between 25,000 and 30,000 visitors from abroad [13].

Another important aspect, due to the characteristics of this population, is the confluence of two pandemics (human immunodeficiency virus (HIV) and monkeypox), which may modify the clinical expression of the latter disease [17].

The objectives of this study were to describe the clinical and epidemiological characteristics of patients with monkeypox infection in this area, and to compare these data in patients with or without previous HIV infection.

## 2. Materials and Methods

All patients with a confirmed diagnosis of monkeypox treated at the Hospital Universitario Insular de Gran Canaria, a tertiary hospital, over 3 months (1 May to 31 July 2022) were studied. All the patients were treated by the UEIMT (Infectious Diseases and Tropical Medicine Unit) and/or the Emergency Department.

The epidemiological data collected included the date of diagnosis, age, gender, country of birth, history of recent travel to endemic areas, sexual orientation (MSM, men who have sex with women, bisexual men, or other), presence of skin lesions in sexual partners and number of sexual partners during the month prior to the date of symptom onset. The clinical data included the following: *(i)* fever (>38°); *(ii)* the detection, location and characteristics of lymph nodes; *(iii)* the presence, number and type of skin lesions; *(iv)* the symptoms of proctitis (rectal pain, tenesmus and/or purulent discharge); *(v)* the detection of pharyngitis; and *(vi)* the presence of complications requiring hospital admission.

Monkeypox diagnosis was made by commercial PCR (RealStar Orthopoxvirus PCR kit^®^, Altona, Singapore) on skin samples. The turnaround time from monkeypox virus testing to result availability was 2 days (IQR: 1–2).

In all the patients, as far as possible, basic screening for other infections was performed, which included at least urethral sampling (for the detection of *Neisseria gonorrhoeae, Chlamydia trachomatis, Mycoplasma genitalium, Mycoplasma hominis, Ureaplasma urealyticum, Ureaplasma parvum* and *Trichomonas vaginalis*) (Allplex STI Essential Assay^®^, Seegene, Seoul, South Korea) and serology (HIV and *Treponema pallidum*) (Alinity Sistem^®^, Abbot, Chicago, IL, USA). In specific cases, rectal and/or pharyngeal samples were taken (for the detection of the same microorganisms as in the urethral exudate, as well as viruses of the *Herpetoviridae* family).

Patients were classified into two groups based on previous HIV infection or not. In HIV-infected individuals, the last viral load and CD4 counts in the three months prior to the monkeypox infection were recorded.

Quantitative data are expressed as the means and ranges, and qualitative data, as percentages. The χ^2^ test and Fisher’s test (where appropriate) were used to assess associations between variables.

## 3. Results

**Figure 1:**
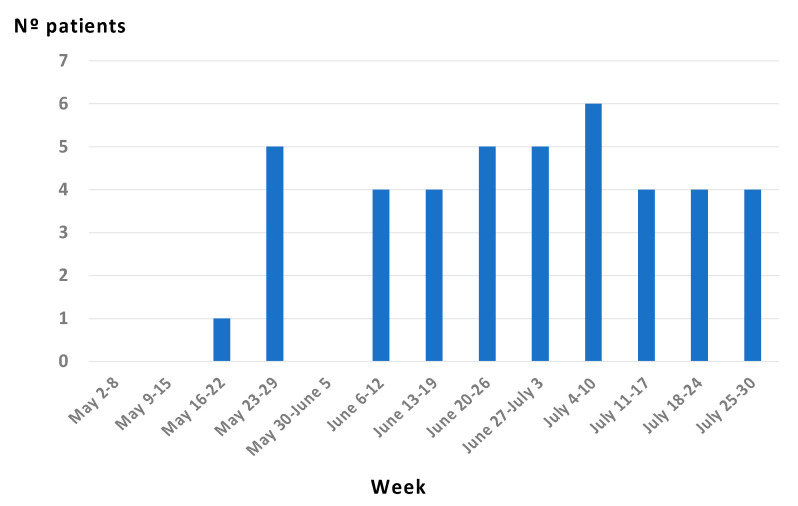
Temporal evolution of PCR–confirmed monkeypox cases.

A total of 42 patients were studied, all of them male. A total of 37/42 reported recent sexual contact with other men, 2/42 reported contact with women, 1 reported bisexual relations, and 2 did not answer this question. Only one patient reported contact with a person with obvious skin lesions. Eighty per cent of the MSM reported three or more sexual contacts in the month prior to the illness that included anonymous sex. Figure 1 shows the dates of the diagnosis of the patients.

The main clinical and epidemiological data, overall and stratified by the history of previous HIV infection, are shown in Table 1. In HIV-infected patients, the mean CD4 count was 759 cells/µL (range: 410–1323) and the viral load was <50 copies/µL in all but two (with viral loads of 68 and 655 copies/µL, respectively).

Most of the patients were in their fourth or fifth decade of life and ranged in age from 22 to 62 years. More than half were foreigners, although none were from or had traveled to endemic areas (Table 1).

All the patients presented one or several nonspecific manifestations (i.e., asthenia, malaise, headache, and/or myalgia) at some point during the illness, although fever was documented in only 36% of them. The fever was moderate (≤38.5°), of short duration (1–3 days) and of variable onset in the course of the disease (both before and after the onset of the rash). Lymphadenopathies, located in either the inguinal or cervical region, were present in 40% of patients. With the exception of two patients (see below), all were of moderate size and without signs of inflammation.

**Figure 2:**
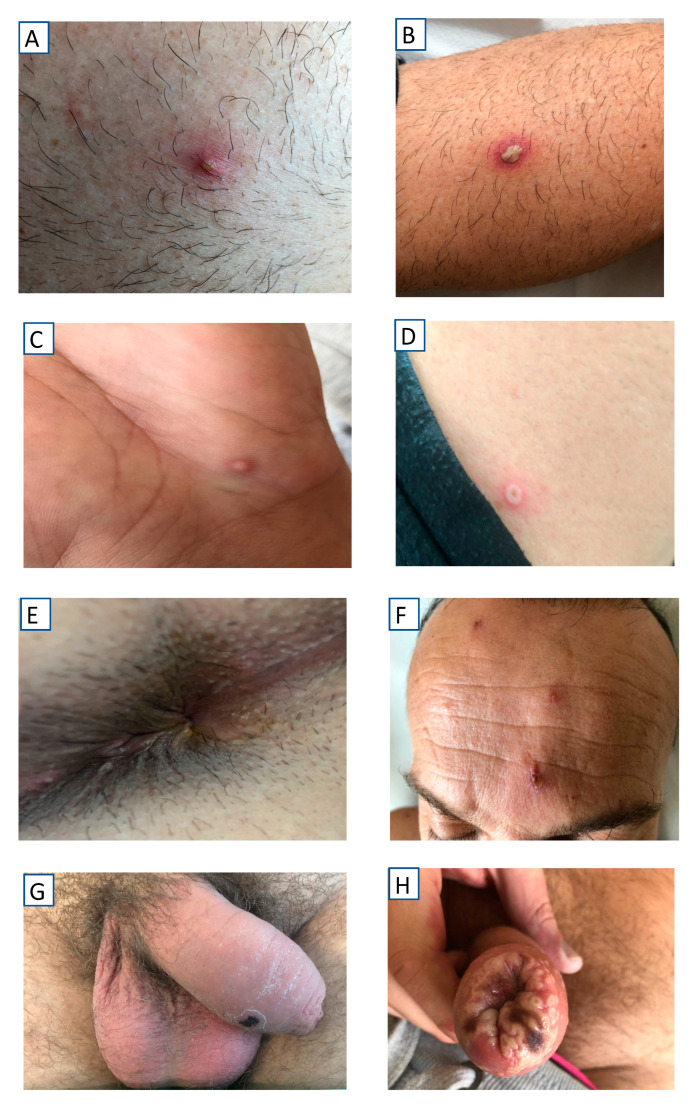
Morphological type of lesions. (**A**) Small pustule (chest); (**B**) Large pustule (leg); (**C**) Papulo-pustule (white center, hand), (**D**) Papulo-pustule (black center, neck); (**E**) Chancriform ulcer (anus); (**F**) Atrophic scar (forehead); (**G**) Necrotic scar (penis); (**H**) Confluent lesions (penis).

**Figure 3:**
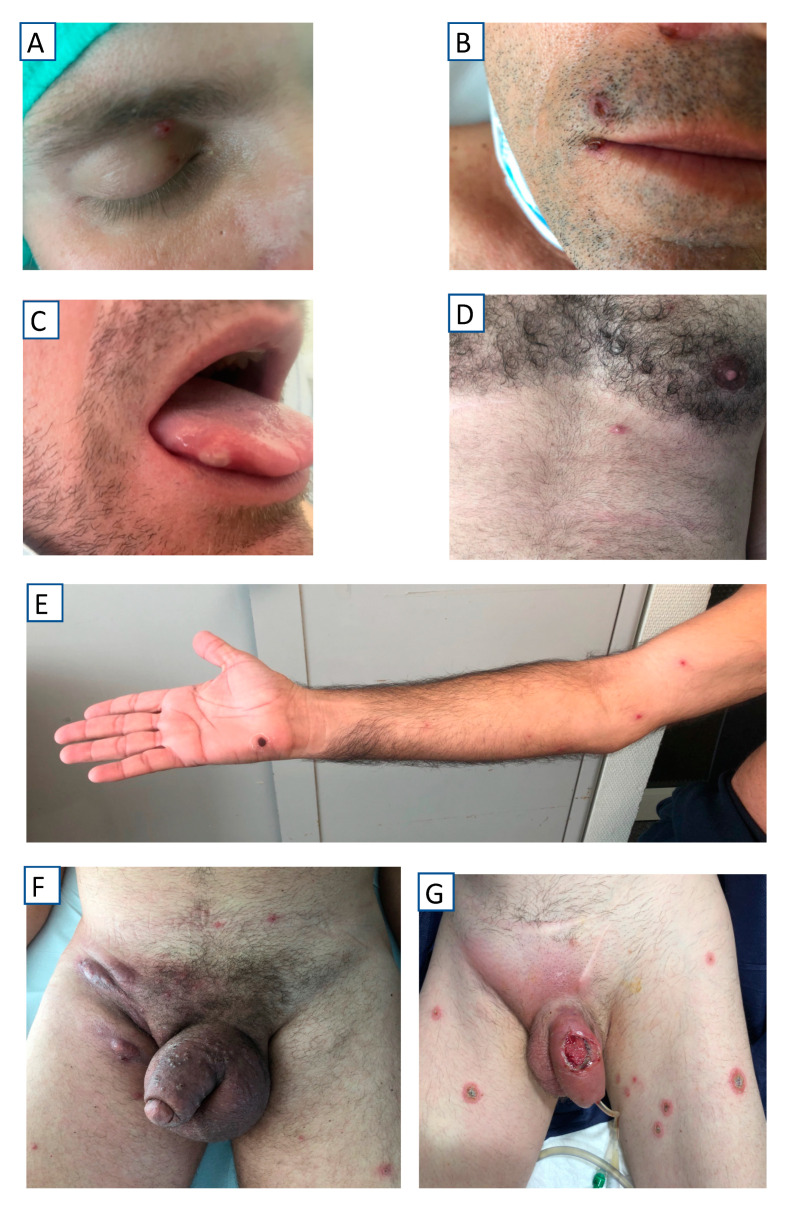
Topographical distribution of lesions. (**A**) Eyelid; (**B**) Perioral; (**C**) Tongue, (**D**) Chest; (**E**) Arm; (**F**) Abdomen, genital area and thighs; (**G**) Abdomen, genital area, and thighs.

**Figure 4:**
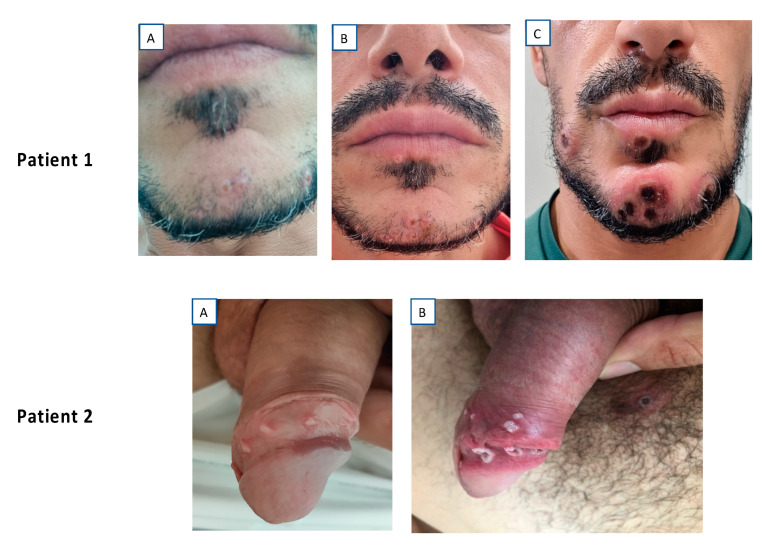
Synchronous evolution of lesions in two patients. Patient 1: (**A**) Day 1; (**B**) Day 3; (**C**) Day 7. Patient 2: (**A**) Day 4; (**B**) Day 8.

All the patients presented mucocutaneous lesions, located as indicated in Table 1. A statistically significant association was observed between oral/perioral involvement and HIV infection. The involvement of palms and soles was only observed in 13% of patients. The number of lesions was highly variable (from a single lesion to more than 25), the lesions occurred in isolation or confluently (Figure 2), and the lesions showed very different morphologic characteristics both between patients and within the same patient (Figure 3). Only in exceptional cases was the synchronous evolution of lesions documented (Figure 4).

## 4. Discussion

On 23 July 2022, the WHO declared monkeypox infection a public health emergency of international concern [18], as it met the requisite criteria: *(i)* an extraordinary event constituting a public health risk, *(ii)* international spread of the disease and *(iii)* potentially requiring a coordinated international response.

The data observed in our series present several differences from previous data on both endemic and imported monkeypox [19] (Table 2).

Thus, incubation periods of 5 to 21 days have been reported [20], although in our experience, it was difficult or impossible to establish it precisely for two reasons. In the first place, only one patient mentioned visible skin lesions on his sexual partner. There are several possible explanations for this: *(i)* the lesions were exclusively mucosal, *(ii)* the number of lesions present was small [14] and *(iii)* the sexual practices did not involve seeing the partner’s skin or genitalia (due to low light in otherwise dark rooms or altered consciousness in chemsex) [11]. At the same time, the number of sexual partners was very variable, making it very difficult to pinpoint the specific incubation time. Considering only the last risky contact, the period ranged between 2 and 35 days. The most common mode of transmission was direct, person-to-person contact (skin and/or mucosal and/or secretions) in the population that had not traveled to endemic areas, both in our series and in the rest of those published during the current outbreak [21]. It is interesting to note that, in the current outbreak, the presence of monkeypox virus has been documented by PCR in various secretions such as saliva, semen, urine, and nasopharyngeal or rectal samples [10,22]. Cutaneous lesions were the most frequent manifestation of monkeypox infection, both in our series and in other published series [8,9,10,11,12,13,14], although their characteristics are difficult to compare due to the method of classification. In terms of morphology, it is possible, although uncommon, for lesions to progress through the traditional evolution from macule to papule to vesicle to pustule to scab. On the other hand, different types of lesions frequently coexist in the same patient (pustules, papules, papulopustules, ulcers or scars), so classification as a single group is often inappropriate. The number of lesions was highly variable, ranging between 1 and 100 per patient, although the usual number was fewer than 25 lesions/patient [23]. Other classic manifestations of monkeypox infection are fever and lymphadenopathy. Fever was present in 36% of the patients, a lower percentage than in other series (52–72%) [8,9,10,11,12,13,14], which means that the absence of this clinical finding does not rule out a diagnosis of monkeypox. The presence of lymphadenopathies was observed in 40% of the patients, at the lower end of the data range indicated by other authors (39–85%) [8,9,10,11,12,13,14]. However, in all cases, the inguinal region was the most frequently affected. Among the “new manifestations” of note in our series were proctitis (12%), pharyngitis (17%) and genital edema (12%). Penile and/or scrotal edema, in particular, have been described in only a few series [10,12]. On the other hand, these new manifestations constituted the most frequent causes of hospital admission in these patients. In summary, the above data reflect a heterogeneous clinical pattern in the current outbreak, with major differences from previous series and several differential nuances compared to other published series. The reasons for some differences may include *(i)* the number of patients and study center (i.e., Reference Sexually Transmitted Infections or Dermatology Services); *(ii)* the age of the patient, since most of the population born before 1972 is vaccinated against smallpox, which exerts a protective effect against monkeypox [11]; and *(iii)* the form of transmission and portal of entry, which result in differences in the viral load present and viral hematogenous spread [10]. However, molecular studies suggest that the strain responsible for the current outbreak is like the one described in West Africa [21], although phylogenetic studies have indicated the possibility of a new lineage [24].

There is much discussion in the literature on whether the current monkeypox outbreak should be considered a sexually transmitted infection (STI) mainly because of the stigma attached to those affected [25]. From a practical point of view, the main mode of transmission of monkeypox in the current outbreak is close personal contact involving skin and/or mucous membranes. Sexual contact includes various activities (kissing, fellatio and anal penetration) that clearly constitute interpersonal contact. It is important for several reasons to bear monkeypox infection in mind in the context of STIs since [26,27,28,29,30] *(i)* a failure to include monkeypox in the differential diagnosis may lead to the overdiagnosis/treatment of other STIs, *(ii)* the overdiagnosis of monkeypox can lead to errors by causing a failure to diagnose other infections (whether sexually transmitted or not) with therapeutic possibilities, and *(iii)* concurrent manifestations of sexually transmitted infections would most likely delay or reduce the possibility of MPXV diagnosis.

In 64% of the patients in the current series, there was a previous diagnosis of HIV infection, which is considerably higher than other published figures (13–44%) [8,9,10,11,12,13,14]. In HIV-infected and immunosuppressed patients, the severity and progression of monkeypox disease are greater [31]. However, in our series, all the coinfected patients had a CD4 count of more than 300 cells per µL. When we compared the clinical characteristics of HIV-positive and -negative patients, we found three main differences: *(i)* a higher frequency of perioral lesions, *(ii)* a higher frequency of pharyngitis and *(iii)* a higher number of infectious transmitted diseases. We speculate that patients with undetectable HIV have a perception of lower risk in sexual relations, specifically in the practice of oral sex.

This study has some limitations. First, all the patients had cutaneous and/or mucosal manifestations, and it is likely, therefore, that paucisymptomatic or asymptomatic individuals were not diagnosed. Second, epidemiological tracing was very difficult because of multiple sexual contacts and high geographic mobility (locals with foreigners, and acquisition by locals on trips to other countries). Indeed, more than half of the patients were foreigners from as many as 16 different countries/nationalities, who were only staying temporarily on the island, which made it difficult, on the one hand, to follow up patients and, on the other, to obtain data on viral load and CD4 in those with a previous diagnosis of HIV.

## 5. Conclusions

In summary, monkeypox is an infectious disease with very varied clinical manifestations that should be considered as a diagnostic possibility in men who have sex with men [32]. Patients coinfected with HIV present a higher frequency of perioral lesions, pharyngitis and STI coinfection. A better understanding of the different characteristics of the current monkeypox outbreak will be useful for implementing preventive measures to combat this infection.

## Figures and Tables

**Table 1:** Demographic and Clinical Characteristics of persons with Monkeypox.

	Total (n = 42)	HIV (n = 27)	Non HIV (n = 15)	*p*
**Age (median; IQR)**	40; 16 (35–51)	45; 16 (36–52)	37: 18 (32–50)	NS
**Origin (n; %)**				NS
***Spanish***	18 (43%)	10/18	8/18	
***Foreign***	24 (57%)	17/24	7/24	
***Europe***	14	9	5	
*Germany*	3	3	0	
*France*	1	0	1	
*United Kingdom*	1	0	1	
*Italy*	3	3	0	
*Poland*	3	1	2	
*Portugal*	1	1	0	
*Russia*	1	0	1	
*Switzerland*	1	1	0	
***Latin America***	9	8	1	
*Argentina*	1	1	0	
*Brazil*	1	1	0	
*Colombia*	1	1	0	
*Cuba*	3	3	0	
*Honduras*	1	1	0	
*Peru*	1	1	0	
*Dominican Republic*	1	0	1	
***Africa***	1	0	1	
*Morocco*	1	0	1	
**Fever (n; %)**	15 (36%)	8/15	7/15	NS
**Lymphadenopathies (n; %)**	17 (40%)	10/17	7/17	NS
***Cervical***	7 (17%)	4/7	3/7	
***Inguinal***	10 (24%)	6/7	4/7	
**Skin and mucosal lessions**	42			
***Head and neck* (n; %)**	25 (60%)	17/25	8/25	**0.01**
*Perioral*	15	12/15	3/15	
*Other*	10	3/10	7/10	
***Trunk* (n; %)**	20 (48%)	13/20	7/25	NS
***Abdomen/Buttocks* (n; %)**	19 (45%)	12/19	7/19	NS
***Genital lesions* (n; %)**	22 (52%)	15/21	7/21	NS
***Perianal* (n; %)**	6 (14%)	3/6	3/6	NS
***Limbs* (n; %)**	30 (71%)	17/29	13/19	NS
*Arms*	14	7/14	7/14	
*Legs*	3	3/3	0/3	
*Both*	13	7/13	6/13	
**Pharyngitis (n; %)**	7 (17%)	7/7	0/7	**0.03**
**Proctitis (n; %)**	5 (12%)	3/5	2/5	NS
**Penis and or scrotal edema (n; %)**	5 (12%)	3/5	2/5	NS
**Local complications (n; %)**	2 (5%)	1/2 Severe dysphagia	1/2 Urinary retention	NS
**Other infectious diseases (n; %)**	11/24 * (50%)	10/11	1/11	**0.03**
	*Chlamydia trachomatis*	1	0	
	*Haemophilus parainfluenzae*	1	0	
	Herpesvirus type 2	1	0	
	MS *Staphylococcus aureus*	1	0	
	*Mycoplasma genitalium*	2	0	
	*Mycoplasma hominis*	1	1	
	*Pantoea septica*	1	0	
	*Streptococcus dysgalactiae (1)*	1	0	
	*Ureaplasma urealyticum (4)*	4	0	

* Three patients have more than one microorganism.

**Table 2:** Comparison of clinical and epidemiological characteristics of the main series of monkeypox infections.

	Present Case Series	Orviz (8)	Girometti (9)	Tarín (10)	Catalá (11)	Patel (12)	Iñigo (13)	Thornhill (14)	*p* *
**Country**	Spain (Canary Islands)	Spain (Madrid)	United Kingdom (London)	Spain (Multicentric)	Spain (Multicentric)	United Kingdom (London)	Spain (Madrid)	International (Multicentric)	-
**Setting**	Infectious & Tropical Service	Reference Sexual Transmitted Infections	Reference Sexual Transmitted Infections	Clinical and University Centers	Dermatology Services	High Consequence Infectious Diseases	Public Health Directorate	-	-
**Patients**	42	48	54	181	185	197	508	528	-
**Median age (years)**	40	35	41	37	38	38	35	38	NS
**MSM (%)**	90	87	100	92	100	99	99	98	NS
**Fever (%)**	36	52	57	72	54	62	64	62	<0.01
**Lymphadenopathies (%)**	40	39	55	85	56	58	61	56	<0.01
**Skin and mucosal lessions**	100	-	100	100	100	100	98	95	NS
*Genital lesions* (%)	52	54	61	55	53	56	72	73	NS
*Perianal* (%)	14	35	44	36	34	41		-	<0.01
**Pharyngitis (%)**	17	-	7	10	-	4.6	28	21	<0.01
**Proctitis (%)**	12	27	-	25	22	17	16	14	NS
**Penile and/or scrotal edema**	12	-	-	8	-	15	-	-	NS
Local complications (%) **	5	2	9	2	2	10	4	13	<0.01
**HIV (%)**	64	39	13	40	42	35.9	44	41	<0.01
**Other infectious diseases (%)**	50 ***	25	-	17	76	32	-	29	<0.01

* χ^2^ test. ** Requiring hospital admission. *** Including STI (sexually transmitted infections) and other infectious diseases.

## Data Availability

Not applicable.
